# Adiposity and risks of colorectal and small intestine cancer in Chinese adults: a prospective study of 0.5 million people

**DOI:** 10.1038/s41416-018-0124-8

**Published:** 2018-06-06

**Authors:** Yuanjie Pang, Christiana Kartsonaki, Yu Guo, Yiping Chen, Ling Yang, Zheng Bian, Fiona Bragg, Iona Y. Millwood, Enke Mao, Yilei Li, Liya Shi, Junshi Chen, Liming Li, Michael V. Holmes, Zhengming Chen

**Affiliations:** 1 Clinical Trial Service Unit & Epidemiological Studies Unit (CTSU), Nuffield Department of Population Health, University of Oxford, Oxford, UK; 2 Medical Research Council Population Health Research Unit (MRC PHRU), Nuffield Department of Population Health, University of Oxford, Oxford, UK; 3 Chinese Academy of Medical Sciences, 9 Dongdan San Tiao, 100730 Beijing, China; 4 Jimai Center for Disease Control and Prevention, 741020 Tianshui, China; 5 Meilan Center for Disease Control and Prevention, 570100 Meilan, China; 6 First Hospital Affiliated to Hainan Medical University, 570102 Haikou, China; 7 National Center for Food Safety Risk Assessment, 37 Guangqu Road, 100021 Beijing, China; 8 School of Public Health, Peking University, 100191 Beijing, China; 9 National Institute for Health Research Oxford Biomedical Research Center, Oxford University Hospital, Old Road, OX3 7LE Oxford, UK

**Keywords:** Risk factors, Colorectal cancer

## Abstract

**Background:**

Uncertainty remains about the associations of adiposity with intestinal cancer in China and by its anatomical subtype.

**Methods:**

The prospective China Kadoorie Biobank recorded 3024 incident cases of colorectal (CRC) and 143 cases of small intestine (SIC) cancer during a 10-year follow-up among 509 568 participants without prior cancer at baseline. Cox regression was used to estimate adjusted hazard ratios (HRs) for specific cancers associated with adiposity.

**Results:**

Overall mean body mass index (BMI) was 23.7  kg/m^2^. BMI was positively associated with CRC (HR per SD 1.10 [95% CI 1.06–1.14]), colon (1.13 [1.07–1.18]), and rectal (1.07 [1.02–1.13]) cancer. For waist circumference, the corresponding HRs per SD were 1.14 (1.10–1.18), 1.18 (1.13–1.24), and 1.11 (1.05–1.16), respectively. The adjusted HRs were somewhat greater in men than women. Adiposity was positively, but non-significantly, associated with SIC risk.

**Conclusions:**

Among relatively lean Chinese adults, adiposity was associated with risks of colon and rectal cancer, with the associations somewhat stronger in men than women.

## Introduction

High body fatness has been established as a cause of colorectal cancer (CRC).^1^ Uncertainty remains, however, as to whether the association differs by sex or by its anatomical site. Moreover, the evidence is rather limited in the East Asian population,^2^ particularly China, where the mean levels of adiposity in the adult population are still much lower than those in the Western population. Despite the low incidence, small intestine cancer (SIC) may arise from the same origin as that of CRC, and therefore may share similar risk factors.^3^ Nonetheless, prospective evidence on the relevance of adiposity for SIC risk has been inconclusive.^4^ The present study examined the associations of general and central adiposity with risks of CRC and SIC within the China Kadoorie Biobank (CKB) population of 0.5 million adults, both overall and by sex and anatomical site.

## Materials and methods

### Study population and follow-up

Overall, 512 891 men and women, aged 30–79 years, were recruited to the CKB from 10 areas in China during 2004–2008.^5^ Extensive data were collected on all participants at baseline through interviewer-administered laptop-based questionnaires, covering socioeconomic and demographic factors, lifestyle habits, personal and family medical history, and current medication. Physical measurements (including anthropometric measures, lung function, heart rate, and blood pressure) and collection of a non-fasting blood sample were undertaken for each participant.

Long-term follow-up data on cause-specific mortality and major morbidity, as well as any episodes of hospitalisation, were collected through linkages via unique national ID number with registries and health insurance databases. The present study excluded participants with a prior history of cancer (*n* = 2577) or missing/implausible adiposity values (*n* = 746), leaving 509 568 participants for the main analysis.

### Exposures and outcomes

Adiposity measures included general (e.g. body mass index [BMI], percent body fat, height adjusted weight) and central adiposity (e.g. waist circumference [WC], waist-to-hip ratio [WHR]). Cancer outcomes included incident cases of CRC (C18–20, *n* = 3024), colon cancer (C18, *n* = 1745), rectal cancer (C20, *n* = 1716), and SIC (C17, *n* = 143) (Supplementary Table S1). Cox regression was used to estimate adjusted hazard ratios (HRs) for specific cancers associated with adiposity, stratified by age-at-risk, sex, and region, and adjusted for age at baseline, education, smoking, alcohol, physical activity, and dietary factors (fresh fruits, vegetables, red meat, and dairy products). A more detailed description of the methods and data source is presented in Supplementary Material.

## Results

Overall mean (SD) BMI and WC were 23.7 (3.3) kg/m^2^ and 80.3 (9.8) cm, respectively. Participants with higher BMI were more likely to have higher systolic blood pressure and random plasma glucose (RPG), and to have prevalent diabetes and a history of cardiovascular disease or hypertension (Supplementary Table S2). The standardised incidence rates of CRC were higher in men and in urban areas (Supplementary Table S3).

There were positive associations of adiposity, irrespective of how it was measured, with the risk of CRC (Supplementary Table S4). For both colon and rectal cancer, the associations of BMI appeared stronger in men than in women (Table 1), although the differences were non-significant (Supplementary Table S5, *p* for heterogeneity = 0.13 and 0.27). Similarly, the positive associations of central adiposity (WC and WHR) were stronger in men than in women (Table 1 and Supplementary Table S5, *p* for heterogeneity = 0.007–0.04). In both sexes, the associations of adiposity with colon cancer appeared stronger than with rectal cancer (Table 1), albeit non-significant (Supplementary Table S5, *p* for heterogeneity = 0.12–0.69). When both BMI and WC were included in the same model (Supplementary Table S6), the risk estimates for WC changed little, while the estimates for BMI attenuated and became non-significant.

**Table 1:** Adjusted HRs of colon and rectal cancer by different measures of adiposity in men and women

Adiposity	Colon						Rectal			
	Men			Women			Men		Women	
	Median^a^	No.^b^	HR (95% CI)^c^	Median	No.	HR (95% CI)	No.	HR (95% CI)	No.	HR (95% CI)
BMI										
<20.0	19.0	100	1.03 (0.84, 1.26)	18.9	90	0.88 (0.71, 1.08)	124	1.08 (0.90, 1.31)	87	0.87 (0.70, 1.07)
20.0 to <22.5	21.3	173	1.00 (0.86, 1.16)	21.4	198	1.00 (0.87, 1.15)	201	1.00 (0.87, 1.15)	188	1.00 (0.87, 1.16)
22.5 to <25.0	23.7	253	1.31 (1.16, 1.48)	23.7	233	0.94 (0.83, 1.07)	237	1.10 (0.97, 1.25)	230	0.99 (0.87, 1.13)
25.0 to <27.5	26.0	188	1.32 (1.14, 1.53)	26.0	195	1.05 (0.91, 1.21)	191	1.23 (1.06, 1.42)	168	0.97 (0.83, 1.12)
≥27.5	28.8	136	1.69 (1.41, 2.01)	29.1	179	1.23 (1.05, 1.43)	134	1.49 (1.24, 1.77)	156	1.12 (0.95, 1.32)
*1-SD increment* ^*d*^			*1.18 (1.10, 1.26)*			*1.10 (1.03, 1.17)*		*1.11 (1.03, 1.19)*		*1.05 (0.98, 1.12)*
WC^e^										
Quintile 1	69.8	131	1.02 (0.85, 1.22)	67.2	131	0.94 (0.79, 1.11)	157	0.98 (0.83, 1.15)	140	1.06 (0.90, 1.26)
Quintile 2	76.0	113	1.00 (0.83, 1.21)	73.4	135	1.00 (0.84, 1.19)	143	1.00 (0.85, 1.18)	126	1.00 (0.84, 1.19)
Quintile 3	81.5	145	1.21 (1.03, 1.42)	78.3	179	1.15 (1.00, 1.34)	167	1.15 (0.99, 1.33)	169	1.17 (1.01, 1.37)
Quintile 4	87.2	212	1.66 (1.45, 1.90)	83.5	207	1.22 (1.06, 1.39)	175	1.15 (0.99, 1.34)	164	1.04 (0.89, 1.21)
Quintile 5	95.0	249	1.86 (1.62, 2.12)	91.5	243	1.23 (1.08, 1.41)	245	1.52 (1.32, 1.74)	230	1.23 (1.07, 1.41)
*1-SD increment* ^*d*^			*1.28 (1.19, 1.37)*			*1.12 (1.04, 1.19)*		*1.18 (1.10, 1.27)*		*1.04 (0.97, 1.12)*
WHR^f^										
Quintile 1	0.82	104	0.75 (0.61, 0.91)	0.78	155	1.22 (1.03, 1.43)	126	0.91 (0.76, 1.09)	107	0.78 (0.64, 0.94)
Quintile 2	0.87	147	1.00 (0.85, 1.18)	0.83	134	1.00 (0.84, 1.19)	149	1.00 (0.85, 1.17)	150	1.00 (0.85, 1.18)
Quintile 3	0.90	141	1.05 (0.89, 1.24)	0.86	131	1.10 (0.93, 1.30)	174	1.30 (1.12, 1.51)	129	0.95 (0.80, 1.12)
Quintile 4	0.93	214	1.28 (1.12, 1.46)	0.89	192	1.24 (1.08, 1.43)	197	1.21 (1.05, 1.39)	202	1.11 (0.96, 1.27)
Quintile 5	0.98	244	1.38 (1.21, 1.57)	0.95	283	1.39 (1.22, 1.57)	241	1.41 (1.24, 1.61)	241	0.96 (0.84, 1.10)
*1-SD increment* ^*d*^			*1.20 (1.14, 1.27)*			*1.07 (1.00, 1.14)*		*1.15 (1.09, 1.23)*		*1.05 (0.98, 1.12)*

*BMI* body mass index, *WC* waist circumference, *WHR* waist-to-hip ratio, *ICD-10 colon* C18, *ICD-10 rectal* C20.

^a^ Median of adiposity measures in each adiposity category.

^b^ Number of cases in each adiposity category.

^c^ Model was stratified by age-at-risk and region, and adjusted for age at baseline, education, smoking, alcohol, physical activity, fresh fruits, vegetables, red meat, and dairy products.

^d^ SD: 3.4 kg/m^2^ for BMI, 9.8 cm for WC, 0.07 for WHR.

^e^ Cut points (cm): men 73.1, 79.0, 84.3, 90.5; women 70.8, 76.0, 81.0, 87.0.

^f^ Cut points: men 0.85, 0.89, 0.92, 0.96; women 0.81, 0.85, 0.88, 0.92

Similarly, hip circumference (HC), percent body fat, height adjusted weight, height, weight-to-height ratio, and weight change, since age 25, all showed positive associations with the risk of CRC, but the association was weaker for BMI at age 25 than for other adiposity measures (Supplementary Table S7). When further adjusting for BMI, the positive associations persisted for WC, HC, and WHR, but not for other measures (Supplementary Figure S1). For these measures of adiposity, the associations were similar for colon and rectal cancer (Supplementary Figure S1), and for proximal and distal colon cancer (Supplementary Table S8).

BMI showed a non-significant, positive trend with the risk of SIC (HR per SD 1.06 [0.89–1.25], Supplementary Table S4). Similarly, there were positive trends for other adiposity traits (Supplementary Table S4 and S7), and the associations were strongest for WC and HC (HR per SD 1.11 [0.94–1.32] and 1.21 [1.01–1.45], respectively).

The associations of adiposity with risks of CRC and SIC changed little when additionally adjusting for diabetes or RPG (Supplementary Table S9), and were also similar in urban and rural areas (Supplementary Table S10).

## Discussion

In this relatively lean Chinese population, we found that general and central adiposity were positively associated with the risk of CRC, with somewhat stronger associations in men and for colon cancer. General and central adiposity showed trends towards higher risk of SIC, in agreement with previous studies in Western population and in Asia (Supplementary Table S11).

The sex- and site-specific findings in the present study are generally consistent with those in previous studies (Supplementary Figure S2). A recent meta-analysis of 38 prospective cohort studies (> 71,000 cases) reported a 6% higher risk of CRC per 5 kg/m^2^ higher BMI,^2^ with somewhat stronger associations in men than women and for colon than rectal cancer.^2^ Despite the much lower mean BMI in China than in North America or Europe,^6^ our risk estimates for BMI appeared somewhat stronger than those in the Western population (per 5 kg/m^2^: 1.14 [1.09–1.21] in CKB vs 1.04 [1.02–1.06] in Europe and 1.05 [1.03–1.07] in North America), but was broadly consistent with those in Asian studies included in that meta-analysis (1.09 [1.01–1.18]).^2^

For WC and WHR, the risk estimates reported in the meta-analysis were somewhat weaker than our estimates.^2^ Moreover, unlike in the present study, they differed little by sex and by anatomical site.^2^ Of all included studies, four studies showed that WC or WHR was more important than BMI in predicting the risk of CRC, while the converse was true for the other two studies.^2^ Our study showed that the association of BMI with CRC risk attenuated when further adjusting for WC, while the association of WC persisted when further controlling for BMI.

The strengths of the CKB include a prospective design, a large and diverse study population, a large number of CRC cases by subtypes, ability to assess a range of adiposity measures, and careful adjustment for other risk factors. Although BMI may not be a good proxy for body fat,^7,8^ we found it is as good as other measures of adiposity, including percent body fat in predicting the risk of CRC. Moreover, we were also able to adjudicate ~30% of all CRC and SIC cancer cases (94% adenocarcinoma, Supplementary Table S12), and showed similar associations for total and adjudicated outcomes (Supplementary Table S13). However, our results do not necessarily indicate causality and residual confounding may still exist.

In conclusion, we showed that both general and central adiposity were associated with the risk of CRC in this relatively lean adult Chinese population. Our study suggested that adiposity, particularly central adiposity, might be associated with higher risk of SIC.

## Electronic supplementary material

Supporting information

## Availability of data and materials

The materials are available upon request; some restrictions will apply.
