# Supplementary material for: Adiposity and risks of colorectal and small intestine cancer in Chinese adults: a prospective study of 0.5 million people
Source: Br J Cancer. 2018 Jun 6;119(2):248–50. doi: 10.1038/s41416-018-0124-8 (PMC6048138; doi:10.1038/s41416-018-0124-8)
Supplement: Supplementary file 1 — Supporting information [file 41416_2018_124_MOESM1_ESM.docx]

**Supplementary Material**

**Adiposity and risks of colorectal and small intestine cancer in Chinese adults:**

**a prospective study of 0.5 million people**

**British Journal of Cancer**

Yuanjie Pang^1^, ScM; Christiana Kartsonaki^1,2^, DPhil; Yu Guo^3^, MSc; Yiping Chen^1,2^, DPhil; Ling Yang^1,2^, PhD; Zheng Bian^3^, MSc; Fiona Bragg^1^, DPhil; Iona Y Millwood^1,2^, DPhil; Enke Mao^4^, BSc; Yilei Li^5^, BSc; Liya Shi^6^, BSc; Junshi Chen^4^, MD; Liming Li^3,5^, MD; Michael V Holmes^1,2,9^, PhD; Zhengming Chen^1^, DPhil

1. Clinical Trial Service Unit & Epidemiological Studies Unit (CTSU), Nuffield Department of Population Health, University of Oxford, Oxford, UK
2. Medical Research Council Population Health Research Unit (MRC PHRU), Nuffield Department of Population Health, University of Oxford, Oxford, UK
3. Chinese Academy of Medical Sciences, 9 Dongdan San Tiao, Beijing 100730, China
4. Jimai Center for Disease Control and Prevention, Tianshui 741020, China
5. Meilan Center for Disease Control and Prevention, Meilan 570100, China
6. First Hospital Affiliated to Hainan Medical University, Haikou 570102, China
7. National Center for Food Safety Risk Assessment, 37 Guangqu Road, Beijing 100021, China
8. School of Public Health, Peking University, Beijing 100191, China
9. National Institute for Health Research Oxford Biomedical Research Centre, Oxford University Hospital, Old Road, Oxford OX3 7LE, UK

**Address for correspondence**

Christiana Kartsonaki

MRC PHRU, CTSU,

Nuffield Department of Population Health

University of Oxford

Oxford, OX3 7LF, UK

Email: christiana.kartsonaki@ndph.ox.ac.uk

Table of Contents

[Supplementary Methods 3](#_Toc510426633)

[Supplementary Table S1. Distribution and classification of colorectal and small intestine cancer by ICD-10 code 7](#_Toc510426634)

[Supplementary Table S2. Participant characteristics by baseline BMI in CKB 8](#_Toc510426635)

[Supplementary Table S3. Standardised incidence rates of colorectal and small intestine cancer by sex and area 9](#_Toc510426636)

[Supplementary Table S4. Adjusted HRs of colorectal and small intestine cancer by different measures of adiposity 10](#_Toc510426637)

[Supplementary Table S5. Adjusted HRs for colon and rectal cancer per 1 SD higher in measures of adiposity in men and women 11](#_Toc510426638)

[Supplementary Table S6. Adjusted HRs for colon and rectal cancer per 1 SD higher in BMI and WC, with and without mutual adjustment in men and women 12](#_Toc510426639)

[Supplementary Table S7. Adjusted HRs of colorectal and small intestine cancer by measures of adiposity and anthropometry 13](#_Toc510426640)

[Supplementary Figure S1. Adjusted HRs for colorectal cancer per 1 SD higher in measures of adiposity, with a) basic adjustment and b) with additional adjustment for BMI, where appropriate 14](#_Toc510426641)

[Supplementary Table S8. Adjusted HRs for colon cancer per 1 SD higher in different measures of adiposity by anatomical subsite 15](#_Toc510426642)

[Supplementary Table S9. Adjusted HRs for colorectal and small intestine cancer per 1 SD higher BMI or WC with additional adjustment for diabetes or random plasma glucose 16](#_Toc510426643)

[Supplementary Table S10. Adjusted HRs for colorectal and small intestine cancer per 1 SD higher BMI or WC in urban and rural areas, separately 17](#_Toc510426644)

[Supplementary Table S11. Selected characteristics of published prospective studies of BMI and small intestine cancer 18](#_Toc510426645)

[Supplementary Figure S2. Comparison of CKB risk estimates with the most recent systematic review and meta-analysis (SRMA) 19](#_Toc510426646)

[Supplementary Table S12. Confirmation status and histological subtypes of colorectal and small intestine cancer 20](#_Toc510426647)

[Supplementary Table S13. Adjusted HRs for colorectal cancer per 1 SD higher in measures of adiposity for total and adjudicated outcomes, separately 21](#_Toc510426648)

[References 22](#_Toc510426649)

# Supplementary Methods

*Study population*

Details of the CKB design, survey methods, and population characteristics have been described elsewhere.^1^ Briefly, 512 891 participants (210 222 men and 302 669 women) aged 30-79 were recruited into the study from 10 (5 urban, 5 rural) localities in China during 2004-2008. The study areas were selected to provide diversity in risk factor exposure and disease patterns, while taking into account population stability, quality of mortality and morbidity registries, capacity, and long-term commitment within the areas. All participants provided written informed consent. Prior international, national and regional ethical approvals were obtained.

*Data collection*

At local study assessment clinics, participants completed an interviewer-administered laptop-based questionnaire on socio-demographic characteristics, smoking, alcohol consumption, diet, physical activity, personal and family medical history and current medication. Personal medical history included history of diabetes, coronary heart disease (CHD), stroke, cancer, and other major conditions. Dietary data covered 12 major food groups: rice, wheat products, other staple foods, red meat, poultry, fish, eggs, dairy products, fresh vegetables, preserved vegetables, fresh fruit, and soybean products. Respondents were asked about the frequency of habitual consumption during the previous 12 months and chose among five categories of frequency (daily, 4 to 6 days per week, 1 to 3 days per week, monthly, or never/rarely). A range of physical measurements were recorded by trained technicians using calibrated instruments with standard protocols. Desktop analysers were used to measure random plasma glucose (RPG).

All anthropometric measurements were taken by trained technicians while participants were wearing light clothes and no shoes, usually to the nearest 0.1 cm or 0.1 kg. Standing height was measured using a stadiometer. Weight was measured using a body composition analyser (TANITA-TBF-300GS; Tanita Corporation), with subtraction of weight of clothing according to season (ranging from 0.5 kg in summer to 2.0-2.5 kg in winter). Waist circumference (WC) and hip circumference (HC) were measured using a soft non-stretchable tape, with HC measured at the maximum circumference around the buttocks.

General adiposity measures were body mass index (BMI), percent body fat (%BF), height adjusted weight, and weight-to-height ratio; central adiposity measures were WC, HC, and waist-to-hip ratio (WHR). BMI at baseline was calculated as the measured weight in kilograms divided by the square of the measured height in metres. BMI at 25 years (BMI25) was calculated using the recalled weight at age 25 years and the measured height at baseline. WHR was the ratio of WC to HC. %BF was the fraction of total weight that was estimated to be fat weight by the Tanita body composition analyser using proprietary algorithms. Weight-to-height ratio was the ratio of weight (kg) to height (m).

*Follow-up for mortality and cancer incidence*

The vital status of each participant was determined periodically through China CDC’s Disease Surveillance Points (DSP) system,^2^ supplemented by regular checks against local residential records and health insurance records and by annual active confirmation through street committees or village administrators. Additional information about cancer incidence and any episodes of hospitalization was collected through linkages, via each participant’s unique national identification number, with disease registries (for cancer, ischemic heart disease, stroke, and diabetes) and national health insurance claims databases, which have almost universal coverage in the study areas. All events were coded using International Classification of Diseases, 10th Revision (ICD-10) by trained staff who were blinded to baseline information.^1^ Information on cancer histological subtypes was also collected for a subset of the cases through cancer registries or reviews of hospital medical notes as part of the ongoing outcome adjudication for major diseases (**Supplementary Table S12**). By 1.1.2016, 42 922 (8%) participants had died, and 5276 (1.0%) were lost to follow-up. Distribution and classification of CRC and SIC are shown in **Supplementary Table S1**.

*Statistical analysis*

The present study excluded individuals with a prior history of cancer (n=2577), extreme values of any adiposity measures (e.g. BMI <15 kg/m^2^ or ≥40 kg/m^2^; n=508) or missing data on BMI or %BF (n=238). After these exclusions, 509 568 individuals remained in the main analyses. Mean values and prevalence of baseline characteristics and incidence rates of CRC and SIC were calculated by BMI categories, using direct standardization to the age (in 5-year groups), sex, and area structure of the population, where appropriate.

Cox regression models were used to estimate adjusted hazard ratios (HRs) of CRC associated with adiposity, stratified by age-at-risk, sex, and study area (10 areas), and adjusted for age at baseline, education (4 groups: no formal school, primary school, middle/high school, or college/university), smoking (3 groups: never regular, former regular, or current regular), alcohol (5 groups: abstainers, ex-weekly drinkers, reduced-intake drinkers, occasional drinkers, or weekly drinkers), total physical activity, and dietary factors (fresh fruits, vegetables, red meat, and dairy products). Dietary factors were modelled using a three-level variable according to intake frequency (daily, weekly, or less than weekly). BMI was modelled as a categorical variable with five categories (<20.0, 20.0 to <22.5, 22.5 to <25.0, 25.0 to <27.5, and ≥27.5 kg/m^2^). BMI was also modelled as a continuous variable to estimate effects per 5 kg/m^2^ or per one standard deviation (SD) increase. For height-adjusted weight, standing height was also included in the model as a continuous variable. Other measures of adiposity were modelled as quintiles and as continuous variables. HRs for each anthropometric category are presented along with the variance of the log risk in each category using so-called ‘floating’ standard errors, so that each HR has a 95% confidence interval (CI) that facilitates comparisons between any two groups.^3^ Statistical analyses were done using SAS version 9.3 and R version 2.14.2.

# Supplementary Table S1. Distribution and classification of colorectal and small intestine cancer by ICD-10 code

| **ICD-10 code** | **Descriptions** | **No. of cases** |
| --- | --- | --- |
| C17.0 | Duodenum | 102 |
| C17.1 | Jejunum | 2 |
| C17.2 | Ileum | 6 |
| C17.3 | Meckel diverticulum | 0 |
| C17.8 | Overlapping lesion of small intestine | 6 |
| C17.9 | Small intestine, unspecified | 29 |
| **C17** | **Small intestine** | **143** |
|  |  |  |
| C18.0 | Caecum | 63 |
| C18.1 | Appendix | 15 |
| C18.2 | Ascending colon | 135 |
| C18.3 | Hepatic flexure | 37 |
| C18.4 | Transverse colon | 45 |
| C18.5 | Splenic flexure | 8 |
| C18.6 | Descending colon | 34 |
| C18.7 | Sigmoid colon | 276 |
| C18.8 | Overlapping lesion of colon | 29 |
| C18.9 | Colon, unspecified | 1474 |
| **C18.0-5** | **Proximal colon** | **294** |
| **C18.6, C18.7** | **Distal colon** | **308** |
| **C18** | **Colon** | **1745** |
|  |  |  |
| **C19** | **Rectosigmoid junction** | **152** |
| **C20** | **Rectum** | **1716** |
|  |  |  |
| **C18-C20** | **Colorectum** | **3024** |

**Supplementary Table S2**. **Participant characteristics by baseline BMI in CKB**

|  | **BMI categories (kg/m^2^)** | | | | | **All** |
| --- | --- | --- | --- | --- | --- | --- |
| **Variable^a^** | **<20.0** | **20.0 to <22.5** | **22.5 to <25.0** | **25.0 to <27.5** | **≥27.5** |  |
|  | (n=65 638) | (n=131 274) | (n=144 745) | (n=101 038) | (n=66 873) | (n=509 568) |
| Age (SD), year | 52.4 (11.8) | 50.7 (10.8) | 51.0 (10.4) | 51.5 (10.2) | 51.7 (10.2) | 51.5 (10.7) |
| Female, % | 55.3 | 57.4 | 59.9 | 59.3 | 63.8 | 59.2 |
| **Socioeconomic and lifestyle factors** |  |  |  |  |  |  |
| Urban region, % | 31.0 | 37.1 | 45.3 | 51.3 | 56 | 44.1 |
| ≥6 years of education, % | 43.3 | 43.9 | 44.1 | 43.8 | 43.1 | 43.4 |
| Household income ≥35 000 RMB/year, % | 23.8 | 24.8 | 25.2 | 25.1 | 24.5 | 24.7 |
| Ever regular smoking, % |  |  |  |  |  |  |
| Male | 76.1 | 71.5 | 65.8 | 62.8 | 61.5 | 67.7 |
| Female | 4.2 | 3.0 | 2.6 | 2.4 | 2.6 | 2.8 |
| Weekly drinking % |  |  |  |  |  |  |
| Male | 31.7 | 34.3 | 33.9 | 32.9 | 32.5 | 33.5 |
| Female | 2.1 | 2.1 | 2.1 | 2.1 | 1.9 | 2.1 |
| Total physical activity (SD), MET h/day | 21.4 (13.9) | 21.8 (14.2) | 21.3 (14.0) | 20.6 (13.6) | 19.8 (13.0) | 21.1 (13.9) |
| **Blood pressure and anthropometry** |  |  |  |  |  |  |
| SBP (SD), mmHg | 123.0 (20.9) | 127.2 (20.4) | 131.3 (20.6) | 134.9 (20.8) | 139.5 (21.4) | 131.1 (21.3) |
| RPG (SD), mmol/L | 5.9 (2.1) | 5.9 (2.1) | 6.0 (2.3) | 6.2 (2.5) | 6.4 (2.7) | 6.1 (2.3) |
| BMI (SD), kg/m^2^ | 18.7 (1.1) | 21.3 (0.7) | 23.7 (0.7) | 25.9 (0.6) | 29.0 (2.0) | 23.7 (3.4) |
| Waist circumference (SD), cm | 68.3 (5.2) | 74.5 (5.3) | 80.6 (5.8) | 86.0 (5.9) | 93.0 (7.4) | 80.3 (9.8) |
| Hip circumference (SD), cm | 83.6 (4.3) | 87.4 (4.2) | 90.9 (4.4) | 94.2 (4.5) | 99.0 (5.9) | 90.9 (6.9) |
| Waist to hip ratio (SD) | 0.82 (0.06) | 0.85 (0.06) | 0.89 (0.06) | 0.91 (0.06) | 0.94 (0.07) | 0.90 (0.07) |
| Body fat percentage (SD), % | 18.9 (4.9) | 23.9 (5.5) | 28.3 (5.8) | 32.1 (6.2) | 36.6 (7.6) | 28.0 (8.4) |
| Height (SD), cm | 158.6 (8.3) | 158.6 (8.1) | 158.7 (8.2) | 158.8 (8.3) | 158.9 (8.5) | 158.7 (8.3) |
| BMI at age 25 (SD), kg/m^2^ | 20.6 (2.3) | 21.4 (2.3) | 22.0 (2.4) | 22.5 (2.5) | 23.2 (2.8) | 22.0 (2.6) |
| **Prior disease history, %** |  |  |  |  |  |  |
| Diabetes | 3.4 | 4.3 | 5.8 | 7.1 | 9.1 | 3.0 |
| Coronary heart disease | 1.9 | 2.2 | 2.9 | 3.5 | 4.3 | 5.9 |
| Stroke or TIA | 1.0 | 1.4 | 1.8 | 2.0 | 2.3 | 1.7 |
| Hypertension | 4.6 | 7.3 | 11.2 | 15.5 | 21.5 | 11.6 |
| Family history of diabetes | 3.7 | 4.3 | 5.0 | 5.4 | 6.0 | 4.9 |
| Family history of cancer | 13.0 | 13.5 | 14.2 | 14.3 | 14.5 | 13.9 |

Abbreviations: BMI, body mass index; MET, metabolic equivalent of task; RPG, random plasma glucose; SBP, systolic blood pressure; TIA, transient ischaemic attack.

^a^ Results were adjusted for age, region, and sex (where appropriate).

# Supplementary Table S3. Standardised incidence rates of colorectal and small intestine cancer by sex and area^a^

| **Cancer type**  **(ICD-10)** | **No. of events** | | **Rate**  **(per 100 000 persons)** | |
| --- | --- | --- | --- | --- |
| **Colorectal (C18-C20)** | | | |  |
| Urban | | 1725 | | 760.7 |
| Male | | 868 | | 944.4 |
| Female | | 857 | | 636.7 |
| Rural | | 1299 | | 442.2 |
| Male | | 643 | | 505.8 |
| Female | | 656 | | 390.5 |
|  | |  | |  |
| **Colon (C18)** | |  | |  |
| Urban | | 1065 | | 470.0 |
| Male | | 516 | | 565.6 |
| Female | | 549 | | 405.6 |
| Rural | | 680 | | 231.9 |
| Male | | 334 | | 263.1 |
| Female | | 346 | | 208.4 |
|  | |  | |  |
| **Rectal (C20)** | |  | |  |
| Urban | | 898 | | 394.3 |
| Male | | 437 | | 514.7 |
| Female | | 425 | | 313.2 |
| Rural | | 818 | | 280.7 |
| Male | | 414 | | 328.8 |
| Female | | 404 | | 241.0 |
|  | |  | |  |
| **Small intestine (C17)** | | | |  |
| Urban | | 77 | | 33.6 |
| Rural | | 66 | | 23.6 |

^a^ Standardised by age, sex, and region (where appropriate).

# Supplementary Table S4. Adjusted HRs of colorectal and small intestine cancer by different measures of adiposity

|  |  | **Colorectal** | | **Colon** | | **Rectal** | | **Small intestine** | |
| --- | --- | --- | --- | --- | --- | --- | --- | --- | --- |
| **Adiposity** | **Median^a^** | **No.^b^** | **HR (95% CI)^c^** | **No.^b^** | **HR (95% CI)^c^** | **No.^b^** | **HR (95% CI)^c^** | **No.^b^** | **HR (95% CI)^c^** |
| **BMI (kg/m^2^)** |  |  |  |  |  |  |  |  |  |
| <20.0 | 18.9 | 349 | 0.95 (0.85, 1.06) | 190 | 0.95 (0.82, 1.10) | 211 | 0.98 (0.85, 1.13) | 16 | 0.88 (0.53, 1.46) |
| 20.0 to <22.5 | 21.3 | 673 | 1.00 (0.93, 1.08) | 371 | 1.00 (0.90, 1.11) | 389 | 1.00 (0.90, 1.11) | 33 | 1.00 (0.71, 1.41) |
| 22.5 to <25.0 | 23.7 | 823 | 1.05 (0.98, 1.12) | 486 | 1.10 (1.01, 1.20) | 467 | 1.05 (0.96, 1.15) | 42 | 1.12 (0.83, 1.51) |
| 25.0 to <27.5 | 26.0 | 646 | 1.10 (1.02, 1.19) | 383 | 1.16 (1.05, 1.29) | 359 | 1.09 (0.98, 1.21) | 28 | 1.04 (0.72, 1.51) |
| ≥27.5 | 29.0 | 533 | 1.32 (1.21, 1.44) | 315 | 1.40 (1.25, 1.57) | 290 | 1.28 (1.13, 1.44) | 24 | 1.37 (0.90, 2.07) |
| *1-SD increment^d^* |  |  | *1.10 (1.06, 1.14)* |  | *1.13 (1.07, 1.18)* |  | *1.07 (1.02, 1.13)* |  | *1.06 (0.89, 1.25)* |
| **WC (cm)** |  |  |  |  |  |  |  |  |  |
| <71.8 | 68.2 | 469 | 0.95 (0.86, 1.04) | 253 | 0.93 (0.82, 1.06) | 283 | 0.98 (0.87, 1.11) | 28 | 1.16 (0.79, 1.71) |
| 71.8 to <77.0 | 74.3 | 454 | 1.00 (0.91, 1.10) | 248 | 1.00 (0.88, 1.13) | 264 | 1.00 (0.89, 1.13) | 21 | 1.00 (0.65, 1.54) |
| 77.0 to <82.2 | 79.5 | 561 | 1.05 (0.97, 1.14) | 319 | 1.08 (0.97, 1.21) | 326 | 1.07 (0.96, 1.19) | 23 | 0.95 (0.64, 1.44) |
| 82.2 to <88.5 | 85.0 | 640 | 1.13 (1.04, 1.22) | 400 | 1.27 (1.16, 1.40) | 331 | 1.02 (0.92, 1.14) | 33 | 1.35 (0.96, 1.90) |
| ≥88.5 | 93.0 | 900 | 1.37 (1.28, 1.47) | 525 | 1.46 (1.34, 1.61) | 512 | 1.36 (1.24, 1.50) | 38 | 1.45 (1.03, 2.04) |
| *1-SD increment^d^* |  |  | *1.14 (1.10, 1.18)* |  | *1.18 (1.13, 1.24)* |  | *1.11 (1.05, 1.16)* |  | *1.11 (0.94, 1.32)* |
| **WHR** |  |  |  |  |  |  |  |  |  |
| <0.82 | 0.79 | 368 | 0.93 (0.84, 1.04) | 224 | 1.01 (0.88, 1.16) | 188 | 0.82 (0.70, 0.94) | 30 | 1.69 (1.16, 2.47) |
| 0.82 to <0.86 | 0.84 | 578 | 1.00 (0.92, 1.09) | 317 | 1.00 (0.89, 1.12) | 348 | 1.00 (0.90, 1.11) | 25 | 1.00 (0.67, 1.48) |
| 0.86 to <0.90 | 0.88 | 507 | 1.15 (1.05, 1.25) | 274 | 1.14 (1.02, 1.29) | 292 | 1.08 (0.96, 1.21) | 24 | 1.30 (0.87, 1.94) |
| 0.90 to <0.94 | 0.91 | 658 | 1.18 (1.09, 1.27) | 382 | 1.26 (1.14, 1.40) | 381 | 1.11 (1.01, 1.23) | 27 | 1.16 (0.80, 1.70) |
| ≥0.94 | 0.97 | 913 | 1.28 (1.20, 1.37) | 548 | 1.42 (1.30, 1.55) | 507 | 1.15 (1.05, 1.26) | 37 | 1.29 (0.92, 1.81) |
| *1-SD increment^d^* |  |  | *1.11 (1.07, 1.14)* |  | *1.12 (1.08, 1.17)* |  | *1.10 (1.05, 1.15)* |  | *0.98 (0.83, 1.17)* |

Abbreviations: BMI, body mass index; WC, waist circumference; WHR, waist-to-hip ratio. ICD-10: colorectal, C18-C20; colon, C18; rectal C20; small intestine C17.

^a^ Median of adiposity measures in each adiposity category.

^b^ Number of cases in each adiposity category.

^c^ Model was stratified by age-at-risk, sex, and region, and adjusted for age at baseline, education, smoking, alcohol, physical activity, fresh fruits, vegetables, red meat, and dairy products.

^d^ SD: 3.4 kg/m^2^ for BMI, 9.8 cm for WC, 0.07 for WHR. For BMI, the HR per SD was 1.05 (0.97-1.14) in the range 15-<25 kg/m^2^ and 1.12 (1.03-1.22) in the range 25-40 kg/m^2^ (*p* for heterogeneity = 0.25).

# Supplementary Table S5. Adjusted HRs for colon and rectal cancer per 1 SD higher in measures of adiposity in men and women

|  | **Colon cancer** |  | **Rectal cancer** |  | *p* for heterogeneity^b^ |
| --- | --- | --- | --- | --- | --- |
|  | **HR (95% CI)^a^** |  | **HR (95% CI)^a^** |  |  |
| **BMI** | |  |  |  |  |
| Male | 1.18 (1.10, 1.26) |  | 1.11 (1.03, 1.19) |  | *0.22* |
| Female | 1.10 (1.03, 1.17) |  | 1.05 (0.98, 1.12) |  | *0.33* |
| *p* for heterogeneity^c^ | *0.13* |  | *0.27* |  |  |
|  |  |  |  |  |  |
| **WC** | |  |  |  |  |
| Male | 1.28 (1.19, 1.37) |  | 1.18 (1.10, 1.27) |  | *0.12* |
| Female | 1.12 (1.04, 1.19) |  | 1.04 (0.97, 1.12) |  | *0.14* |
| *p* for heterogeneity^c^ | *0.007* |  | *0.01* |  |  |
|  |  |  |  |  |  |
| **WHR** | |  |  |  |  |
| Male | 1.20 (1.14, 1.27) |  | 1.15 (1.09, 1.23) |  | *0.31* |
| Female | 1.07 (1.00, 1.14) |  | 1.05 (0.98, 1.12) |  | *0.69* |
| *p* for heterogeneity^c^ | *0.008* |  | *0.04* |  |  |

Abbreviations: BMI, body mass index; WC, waist circumference; WHR, waist-to-hip ratio.

ICD-10: colon, C18; rectal C20.

SD: 3.4 kg/m^2^ for BMI, 9.8 cm for WC, 0.07 for WHR.

^a^ Model was stratified by age-at-risk and region, and adjusted for age at baseline, education, smoking,

alcohol, physical activity, fresh fruits, vegetables, red meat, and dairy products.

^b^ *p* for heterogeneity by anatomical site.

^c^ *p* for heterogeneity by sex.

# Supplementary Table S6. Adjusted HRs for colon and rectal cancer per 1 SD higher in BMI and WC, with and without mutual adjustment in men and women^a^

|  | **Overall** | |  | **Male** | |  | **Female** | |
| --- | --- | --- | --- | --- | --- | --- | --- | --- |
|  | **Colon** | **Rectal** |  | **Colon** | **Rectal** |  | **Colon** | **Rectal** |
|  | **HR (95% CI)** | **HR (95% CI)** |  | **HR (95% CI)** | **HR (95% CI)** |  | **HR (95% CI)** | **HR (95% CI)** |
| **BMI** |  |  |  |  |  |  |  |  |
| Basic | 1.13 (1.07, 1.18) | 1.07 (1.02, 1.13) |  | 1.18 (1.10, 1.26) | 1.11 (1.03, 1.19) |  | 1.10 (1.03, 1.17) | 1.05 (0.98, 1.12) |
| + WC | 0.90 (0.82, 0.99) | 0.92 (0.84, 1.01) |  | 0.79 (0.68, 0.92) | 0.80 (0.70, 0.93) |  | 0.98 (0.87, 1.10) | 1.02 (0.90, 1.15) |
|  |  |  |  |  |  |  |  |  |
| **WC** |  |  |  |  |  |  |  |  |
| Basic | 1.18 (1.13, 1.24) | 1.11 (1.05, 1.16) |  | 1.28 (1.19, 1.37) | 1.18 (1.10, 1.27) |  | 1.12 (1.04, 1.19) | 1.04 (0.97, 1.12) |
| + BMI | 1.30 (1.19, 1.43) | 1.20 (1.10, 1.32) |  | 1.56 (1.36, 1.80) | 1.43 (1.25, 1.64) |  | 1.15 (0.87, 1.31) | 1.04 (0.91, 1.18) |

Abbreviations: BMI, body mass index; WC, waist circumference. SD: 3.4 kg/m^2^ for BMI, 9.8 cm for WC.

ICD-10: colon, C18; rectal C20.

^a^ Model was stratified by age-at-risk, sex, and region, and adjusted for age at baseline, education, smoking, alcohol, physical activity, fresh fruits, vegetables, red meat, and

dairy products.

# Supplementary Table S7. Adjusted HRs of colorectal and small intestine cancer by measures of adiposity and anthropometry

|  |  | **Colorectal** | | **Small intestine** | |
| --- | --- | --- | --- | --- | --- |
| **Adiposity** | **Median^a^** | **No.^b^** | **HR (95% CI)^c^** | **No.^b^** | **HR (95% CI)^c^** |
| **Hip circumference (cm)** | |  |  |  |  |
| <85.1 | 82.5 | 559 | 0.92 (0.84, 1.00) | 22 | 0.66 (0.42, 1.03) |
| 85.1 to <89.0 | 87.0 | 530 | 1.00 (0.92, 1.09) | 29 | 1.00 (0.69, 1.45) |
| 89.0 to <92.2 | 90.5 | 555 | 1.03 (0.95, 1.12) | 29 | 1.01 (0.70, 1.45) |
| 92.2 to <96.4 | 94.0 | 594 | 1.07 (0.99, 1.16) | 27 | 0.98 (0.67, 1.43) |
| ≥96.4 | 99.9 | 786 | 1.23 (1.14, 1.34) | 36 | 1.31 (0.90, 1.89) |
| *1-SD increment^d^* |  |  | *1.11 (1.07, 1.16)* |  | *1.21 (1.01, 1.45)* |
| **Percent body fat (%)** |  |  |  |  |  |
| <20.5 | 17.0 | 633 | 0.85 (0.77, 0.94) | 29 | 0.85 (0.55, 1.32) |
| 20.5 to <25.5 | 23.1 | 658 | 1.00 (0.92, 1.08) | 30 | 1.00 (0.69, 1.45) |
| 25.5 to <30.0 | 27.6 | 572 | 1.02 (0.94, 1.10) | 24 | 0.92 (0.62, 1.37) |
| 30.0 to <35.0 | 32.2 | 538 | 1.04 (0.95, 1.14) | 41 | 1.72 (1.23, 2.40) |
| ≥35.0 | 38.8 | 622 | 1.14 (1.04, 1.26) | 19 | 0.80 (0.49, 1.31) |
| *1-SD increment^d^* |  |  | *1.09 (1.04, 1.14)* |  | *1.09 (0.89, 1.35)* |
| **Height-adjusted weight (kg)** | |  |  |  |  |
| <50.7 | 46.9 | 539 | 0.93 (0.85, 1.03) | 26 | 1.44 (0.93, 2.21) |
| 50.7 to <56.2 | 53.5 | 542 | 1.00 (0.92, 1.09) | 25 | 1.00 (0.64, 1.56) |
| 56.2 to <61.5 | 58.7 | 549 | 1.01 (0.93, 1.09) | 27 | 1.52 (1.07, 2.14) |
| 61.5 to <68.4 | 64.5 | 593 | 1.03 (0.95, 1.11) | 31 | 1.43 (1.00, 2.03) |
| ≥68.4 | 74.1 | 801 | 1.30 (1.19, 1.42) | 34 | 1.37 (0.90, 2.09) |
| *1-SD increment^d^* |  |  | *1.13 (1.08, 1.18)* |  | *1.08 (0.87, 1.33)* |
| **Weight-to-height ratio (kg/m)** | |  |  |  |  |
| <0.061 | 0.058 | 522 | 0.98 (0.89, 1.07) | 26 | 1.03 (0.69, 1.55) |
| 0.061 to <0.066 | 0.063 | 523 | 1.00 (0.92, 1.09) | 25 | 1.00 (0.67, 1.48) |
| 0.066 to <0.072 | 0.068 | 543 | 0.99 (0.91, 1.08) | 27 | 1.04 (0.71, 1.51) |
| 0.072 to < 0.079 | 0.077 | 636 | 1.08 (1.00, 1.17) | 31 | 1.14 (0.80, 1.61) |
| ≥0.079 | 0.082 | 800 | 1.27 (1.18, 1.37) | 34 | 1.22 (0.85, 1.74) |
| *1-SD increment^d^* |  |  | *1.10 (1.06, 1.14)* |  | *1.08 (0.90, 1.28)* |
| **Height (cm)** |  |  |  |  |  |
| <151.5 | 148.4 | 593 | 0.97 (0.87, 1.07) | 25 | 0.80 (0.49, 1.31) |
| 151.5 to <156.0 | 153.8 | 526 | 1.00 (0.91, 1.10) | 25 | 1.00 (0.65, 1.54) |
| 156.0 to < 160.5 | 158.1 | 601 | 1.08 (1.00, 1.16) | 26 | 1.11 (0.76, 1.61) |
| 160.5 to <166.0 | 163.0 | 632 | 1.08 (0.99, 1.18) | 28 | 1.29 (0.86, 1.93) |
| ≥166.0 | 169.8 | 672 | 1.13 (1.02, 1.25) | 39 | 1.86 (1.16, 2.96) |
| *1-SD increment^d^* |  |  | *1.06 (1.01, 1.12)* |  | *1.16 (0.90, 1.49)* |
| **BMI at age 25 (kg/m^2^)** |  |  |  |  |  |
| <20.0 | 19.0 | 484 | 0.87 (0.79, 0.95) | 23 | 0.94 (0.62, 1.43) |
| 20.0 to <22.5 | 21.3 | 1021 | 1.00 (0.94, 1.06) | 47 | 1.00 (0.75, 1.33) |
| 22.5 to <25.0 | 23.5 | 725 | 1.00 (0.93, 1.08) | 32 | 0.99 (0.70, 1.40) |
| 25.0 to <27.5 | 25.9 | 246 | 0.94 (0.83, 1.07) | 13 | 1.17 (0.68, 2.04) |
| ≥27.5 | 28.6 | 90 | 1.06 (0.86, 1.30) | 3 | 0.87 (0.28, 2.72) |
| *1-SD increment^d^* |  |  | *1.04 (1.00, 1.08)* |  | *1.10 (0.92, 1.31)* |
| **Weight change since age 25 (kg)** | |  |  |  |  |
| <-2.7 | -6.3 | 558 | 1.00 (0.92, 1.10) | 25 | 0.91 (0.62, 1.35) |
| -2.7 to <2.1 | -0.1 | 439 | 1.00 (0.91, 1.10) | 28 | 1.00 (0.68, 1.48) |
| 2.1 to <6.7 | 4.4 | 444 | 1.02 (0.93, 1.11) | 22 | 0.90 (0.59, 1.37) |
| 6.7 to <12.1 | 9.1 | 482 | 1.02 (0.94, 1.12) | 21 | 0.83 (0.54, 1.28) |
| ≥12.1 | 16.5 | 643 | 1.21 (1.11, 1.31) | 22 | 0.81 (0.53, 1.25) |
| *1-SD increment^d^* |  |  | *1.06 (1.02, 1.10)* |  | *0.98 (0.81, 1.17)* |

ICD-10: colorectal, C18-C20; small intestine C17.

^a^ Median of adiposity measures in each adiposity category.

^b^ Number of cases in each adiposity category.

^c^ Model was stratified by age-at-risk, sex, and region, and adjusted for age at baseline, education, smoking, alcohol, physical activity, fresh fruits, vegetables, red meat, and dairy products.

^d^ SD: 6.9 cm for hip circumference, 8.4% for percent body fat, 10.8 kg for weight, 0.01 for weight-to-height ratio, 8.3 cm

for height, 2.6 kg/m^2^ for BMI at age 25, 9.1 kg for weight change since age 25.

# Supplementary Figure S1. Adjusted HRs for colorectal cancer per 1 SD higher in measures of adiposity, with a) basic adjustment and b) with additional adjustment for BMI, where appropriate


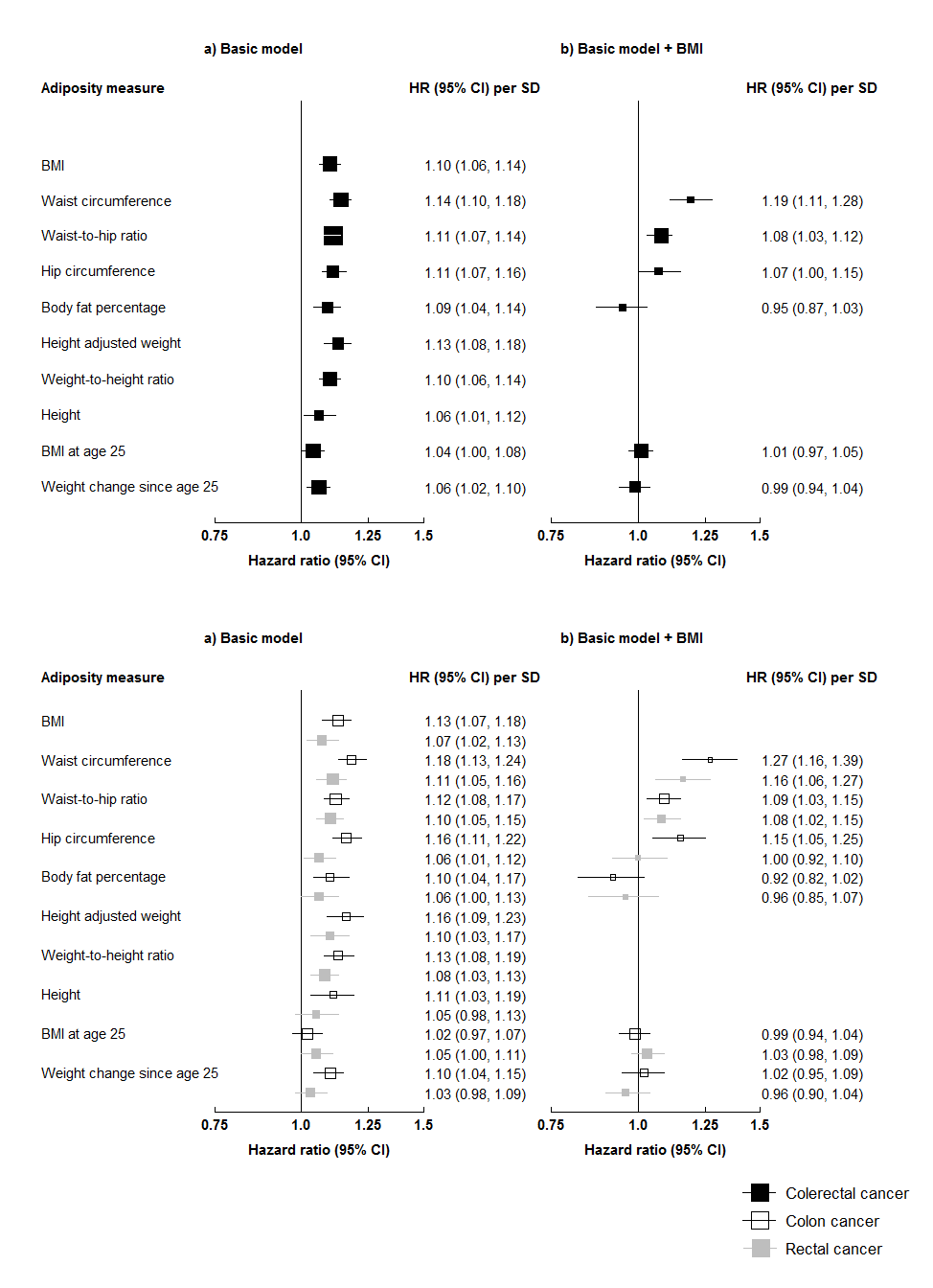


# Supplementary Table S8. Adjusted HRs for colon cancer per 1 SD higher in different measures of adiposity by anatomical subsite^a^

| **Adiposity** | **Total colon** | **Proximal colon** | **Distal colon** | **Distal colon + rectal** |
| --- | --- | --- | --- | --- |
|  | **HR (95% CI)** | **HR (95% CI)** | **HR (95% CI)** | **HR (95% CI)** |
| No. of cases | 1745 | 294 | 308 | 2044 |
| BMI | 1.13 (1.07, 1.18) | 1.23 (1.10, 1.38) | 1.14 (1.01, 1.27) | 1.08 (1.03, 1.13) |
| WC | 1.18 (1.13, 1.24) | 1.27 (1.13, 1.43) | 1.21 (1.08, 1.36) | 1.12 (1.07, 1.17) |
| WHR | 1.12 (1.08, 1.17) | 1.15 (1.04, 1.27) | 1.13 (1.04, 1.24) | 1.11 (1.06, 1.16) |
| BMI25 | 1.02 (0.97, 1.07) | 1.08 (0.96, 1.22) | 0.97 (0.86, 1.09) | 1.03 (0.99, 1.08) |

Abbreviations: BMI, body mass index; WC, waist circumference; WHR, waist-to-hip ratio; BMI25, BMI at age 25.

SD: 3.4 kg/m^2^ for BMI, 9.8 cm for WC, 0.07 for WHR, 2.6 kg/m^2^ for BMI at age 25.

ICD-10: total colon, C18; proximal colon, C18.0-5; distal colon, C18.6, C18.7; distal colon + rectal, C18.6, C18.7, C19, C20.

^a^ Model was stratified by age-at-risk, sex, and region, and adjusted for age at baseline, education, smoking, alcohol, physical activity, fresh fruits, vegetables, red meat,

and dairy products.

*p* for heterogeneity comparing proximal and distal colon cancer: BMI, 0.35; WC, 0.57; WHR, 0.70; BMI25, 0.26.

# Supplementary Table S9. Adjusted HRs for colorectal and small intestine cancer per 1 SD higher BMI or WC with additional adjustment for diabetes or random plasma glucose

|  | **HR (95% CI) per SD^a^** | **HR (95% CI) per SD^a^** |
| --- | --- | --- |
|  | **BMI** | **WC** |
| **Colorectal** |  |  |
| Basic model | 1.10 (1.06, 1.14) | 1.14 (1.10, 1.18) |
| + diabetes | 1.09 (1.05, 1.14) | 1.13 (1.09, 1.18) |
| + RPG | 1.09 (1.05, 1.13) | 1.13 (1.08, 1.17) |
| **Colon** |  |  |
| Basic model | 1.13 (1.07, 1.18) | 1.18 (1.13, 1.24) |
| + diabetes | 1.12 (1.07, 1.18) | 1.19 (1.13, 1.25) |
| + RPG | 1.12 (1.07, 1.17) | 1.19 (1.13, 1.25) |
| **Rectal** |  |  |
| Basic model | 1.07 (1.02, 1.13) | 1.11 (1.05, 1.16) |
| + diabetes | 1.07 (1.02, 1.13) | 1.11 (1.06, 1.17) |
| + RPG | 1.06 (1.01, 1.12) | 1.10 (1.05, 1.16) |
| **Small intestine** |  |  |
| Basic model | 1.06 (0.89, 1.25) | 1.11 (0.94, 1.32) |
| + diabetes | 1.07 (0.90, 1.27) | 1.13 (0.95, 1.34) |
| + RPG | 1.08 (0.90, 1.28) | 1.14 (0.96, 1.36) |

Abbreviations: BMI, body mass index; WC, waist circumference; RPG, random plasma glucose.

SD: 3.4 kg/m^2^ for BMI, 9.8 cm for WC.

ICD-10: colorectal, C18-C20; colon, C18; rectal C20; small intestine C17.

^a^ Basic model was stratified by age-at-risk, sex, and region, and adjusted for age at baseline,

education, smoking, alcohol, physical activity, fresh fruits, vegetables, red meat, and dairy products.

# Supplementary Table S10. Adjusted HRs for colorectal and small intestine cancer per 1 SD higher BMI or WC in urban and rural areas, separately

|  |  | **HR (95% CI) per SD^a^** | **HR (95% CI) per SD^a^** |
| --- | --- | --- | --- |
|  | **No.** | **BMI** | **WC** |
| **Colorectal** |  |  |  |
| Urban | 1299 | 1.09 (1.04, 1.15) | 1.11 (1.06, 1.17) |
| Rural | 1725 | 1.10 (1.04, 1.16) | 1.16 (1.10, 1.23) |
| *p* for heterogeneity |  | *0.81* | *0.24* |
| **Colon** |  |  |  |
| Urban | 680 | 1.13 (1.06, 1.20) | 1.17 (1.10, 1.25) |
| Rural | 1065 | 1.12 (1.03, 1.21) | 1.21 (1.12, 1.31) |
| *p* for heterogeneity |  | *0.86* | *0.51* |
| **Rectal** |  |  |  |
| Urban | 818 | 1.06 (0.99, 1.13) | 1.09 (1.02, 1.17) |
| Rural | 898 | 1.09 (1.01, 1.17) | 1.15 (1.07, 1.23) |
| *p* for heterogeneity |  | *0.58* | *0.28* |
| **Small intestine** |  |  |  |
| Urban | 66 | 0.96 (0.76, 1.21) | 1.03 (0.81, 1.30) |
| Rural | 77 | 1.20 (0.94, 1.54) | 1.22 (0.96, 1.57) |
| *p* for heterogeneity |  | *0.19* | *0.33* |

Abbreviations: BMI, body mass index; WC, waist circumference. SD: 3.4 kg/m^2^ for BMI, 9.8 cm for WC.

ICD-10: colorectal, C18-C20; colon, C18; rectal, C20; small intestine, C17.

^a^ Model was stratified by age-at-risk, sex, and region, and adjusted for age at baseline, education,

smoking, alcohol, physical activity, fresh fruits, vegetables, red meat, and dairy products.

# Supplementary Table S11. Selected characteristics of published prospective studies of BMI and small intestine cancer

| **Author, publication year, country/ region** | **Study name** | **Follow-up period, median/mean** | **Study size, number of cases** | **Assessment of weight and height** | **Adiposity measure** | **Description of categories** | **RR (95% CI)** | **Adjustment for confounders** |
| --- | --- | --- | --- | --- | --- | --- | --- | --- |
| Lu, 2016, Europe^4^ | European Prospective Investigation into Cancer and Nutrition | 1992-2010, 13.9 years | 521 330, 131 cases | Self-reported and measured | BMI  Waist circumference  Hip circumference  Waist-to-hip ratio  Waist-to-height ratio | Per 5 kg/m^2^  Per 5 cm  Per 5 cm  Per 1%  Per 1% | 1.00 (0.94, 1.05)  1.04 (0.94, 1.16)  1.09 (0.93, 1.28)  1.02 (0.99, 1.05)  1.00 (0.97, 1.04) | Age, sex, country, education, smoking, alcohol, physical activity |
| Cross, 2013, US^5^ | National Institutes of Cancer-AARP Diet and Health Study | 1995-2006, 10.5 years | 498 376,  237 cases | Self-reported | BMI | 18.5-<25  25-<30  30-<35  ≥35 | 1.00  1.06 (0.78, 1.44)  0.96 (0.63, 1.45)  1.77 (1.11, 2.82) | Age, sex, follow-up time |
| Boffetta, 2012, Asia^6^ | Asia Cohort Consortium | 1990s-2006, 10.6 years | 0.5M,  134 cases | Self-reported and measured | BMI | <20  20-<22.5  22.5-<25  25-<27.5  ≥27.5 | 0.87 (0.45, 1.67)  1.47 (0.90, 2.38)  1.00  1.49 (0.85, 2.59)  1.50 (0.76, 2.96) | Age, sex, education, alcohol |
| Samanic, 2006, Sweden^7^ | Cohort of Swedish Men | 1971-1999, 13.3 years | 362 552 men, 139 cases | Measured | BMI | 18.5-<25  25-<30  ≥30 | 1.00  1.44 (1.01, 2.04)  1.16 (0.58, 2.36) | Age, year, smoking |
| Bjorge, 2005, Norway^8^ | Cohort of Norwegians | 1960s-2001, 23 years | 2M, 1162 cases | Measured | BMI | M: <18.5  18.5-<25  25-<30  ≥30  F: <18.5  18.5-<25  25-<30  ≥30 | 1.53 (0.57, 4.11)  1.00  1.22 (1.03, 1.45)  1.59 (1.13, 2.23)  1.20 (0.64, 2.25)  1.00  0.94 (0.78, 1.13)  0.98 (0.76, 1.26) | Age, birth cohort |
| Samanic, 2004, US^9^ | Cohort of US veterans | 1969-1996, 27 years | 4.5 M men, 758 cases | Hospitalisation for obesity | BMI | White: ≥30 vs <30  Black: ≥30 vs <30 | 1.58 (1.18, 2.12)  1.07 (0.54, 2.08) | Age, calendar year |

Abbreviations: BMI, body mass index; F, female; M, male.

# Supplementary Figure S2. Comparison of CKB risk estimates with the most recent systematic review and meta-analysis (SRMA)


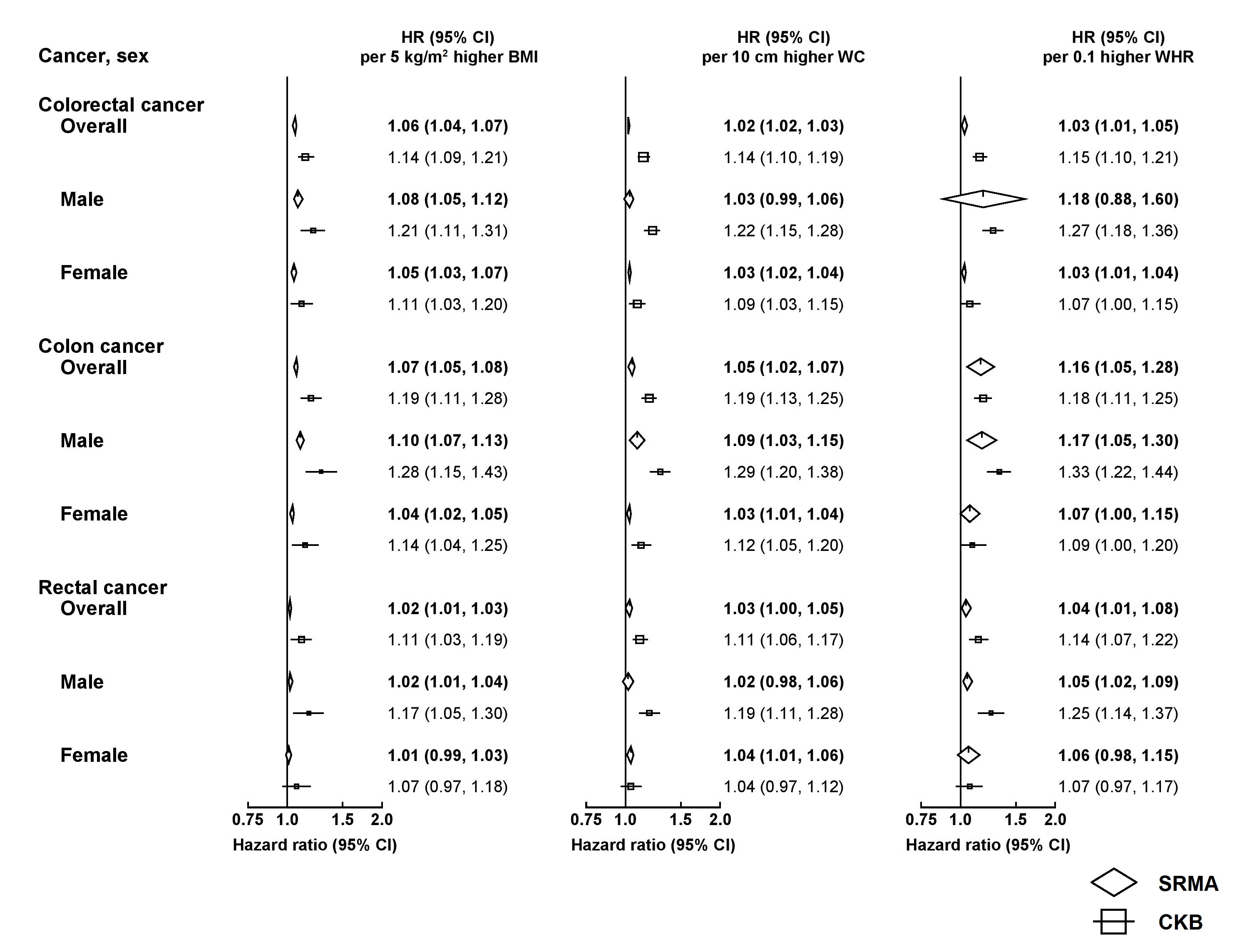


# Supplementary Table S12. Confirmation status and histological subtypes of colorectal and small intestine cancer

|  | **Total** | **Confirmation status** | | | **Histological subtype** | | |
| --- | --- | --- | --- | --- | --- | --- | --- |
|  |  | **Confirmed** | **Refuted** | **Unknown^a^** | **Adenocarcinoma** | **Other** | **Unknown^a^** |
| **Small intestine** | 20 | 12 | 3 | 5 | 5 | 2 | 2 |
| **Colon** | 444 | 355 | 15 | 74 | 275 | 8 | 13 |
| **Proximal colon** | 50 | 41 | 1 | 8 | 37 | 2 | 0 |
| **Distal colon** | 53 | 41 | 0 | 12 | 38 | 0 | 3 |
| **Rectal** | 453 | 351 | 14 | 88 | 270 | 5 | 11 |
| **Colorectal** | 909 | 716 | 29 | 164 | 552 | 14 | 24 |

ICD-10: small intestine, C17; colon, C18; proximal colon, C18.0-5; distal colon, C18.6, C18.7; rectal C20; colorectal C18-C20.

^a^ Unknown status: unable to retrieve medical notes or only admission records were available.

# Supplementary Table S13. Adjusted HRs for colorectal cancer per 1 SD higher in measures of adiposity for total and adjudicated outcomes, separately^a^

|  | **Colon** | **Proximal colon** | **Distal colon** | **Distal colon+rectum** | **Rectum** | **Colorectum** | **Small intestine** |
| --- | --- | --- | --- | --- | --- | --- | --- |
|  | **HR (95% CI)** | **HR (95% CI)** | **HR (95% CI)** | **HR (95% CI)** | **HR (95% CI)** | **HR (95% CI)** | **HR (95% CI)** |
| **Total** |  |  |  |  |  |  |  |
| No. of cases | 1745 | 294 | 308 | 2044 | 1716 | 3024 | 134 |
| BMI | 1.13 (1.07, 1.18) | 1.23 (1.10, 1.38) | 1.14 (1.01, 1.27) | 1.08 (1.03, 1.13) | 1.07 (1.02, 1.13) | 1.10 (1.06, 1.14) | 1.06 (0.89, 1.25) |
| WC | 1.18 (1.13, 1.24) | 1.27 (1.13, 1.43) | 1.21 (1.08, 1.36) | 1.12 (1.07, 1.17) | 1.11 (1.05, 1.16) | 1.14 (1.10, 1.18) | 1.11 (0.94, 1.32) |
| WHR | 1.12 (1.08, 1.17) | 1.15 (1.04, 1.27) | 1.13 (1.04, 1.24) | 1.11 (1.06, 1.16) | 1.10 (1.05, 1.15) | 1.11 (1.07, 1.14) | 0.98 (0.83, 1.17) |
|  |  |  |  |  |  |  |  |
| **Adjudicated** |  |  |  |  |  |  |  |
| No. of cases | 355 | 41 | 41 | 392 | 351 | 716 | 12 |
| BMI | 1.17 (1.06, 1.28) | 1.09 (0.82, 1.45) | 1.09 (0.83, 1.44) | 1.08 (0.99, 1.18) | 1.07 (0.98, 1.18) | 1.12 (1.05, 1.20) | 1.34 (0.97, 2.08) |
| WC | 1.23 (1.11, 1.35) | 1.18 (0.89, 1.56) | 1.19 (0.90, 1.57) | 1.12 (1.02, 1.22) | 1.11 (1.00, 1.22) | 1.17 (1.09, 1.25) | 1.16 (0.74, 1.84) |
| WHR | 1.16 (1.07, 1.25) | 1.08 (0.81, 1.43) | 1.17 (0.98, 1.40) | 1.10 (1.01, 1.20) | 1.08 (0.98, 1.19) | 1.12 (1.06, 1.20) | 1.06 (0.46, 1.20) |

Abbreviation: BMI, body mass index; WC, waist circumference; WHR, waist-to-hip ratio. SD: 3.4 kg/m^2^ for BMI, 9.8 cm for WC, 0.07 for WHR.

ICD-10: total colon, C18; proximal colon, C18.0-5; distal colon, C18.6, C18.7; distal colon + rectal, C18.6, C18.7, C19, C20; rectal, C20; colorectal, C18-C20; small intestine, C17.

**^a^** Model was stratified by age-at-risk, sex, and region, and adjusted for age at baseline, education, smoking, alcohol, physical activity, fresh fruits, vegetables, red meat, and dairy products.

# References

1. Chen Z, Chen J, Collins R, Guo Y, Peto R, Wu F, et al. China Kadoorie Biobank of 0.5 million people: survey methods, baseline characteristics and long-term follow-up. *Int J Epidemiol* 2011;40(6):1652-66.

2. Yang G, Rao C, Ma J, Wang L, Wan X, Dubrovsky G, et al. Validation of verbal autopsy procedures for adult deaths in China. *Int J Epidemiol* 2005;35(3):741-48.

3. Easton DF, Peto J, Babiker AG. Floating absolute risk: an alternative to relative risk in survival and case‐control analysis avoiding an arbitrary reference group. *Stat Med* 1991;10(7):1025-35.

4. Lu Y, Cross AJ, Murphy N, Freisling H, Travis RC, Ferrari P, et al. Comparison of abdominal adiposity and overall obesity in relation to risk of small intestinal cancer in a European Prospective Cohort. *Cancer Causes Control* 2016;27(7):919-27.

5. Cross AJ, Hollenbeck AR, Park Y. A large prospective study of risk factors for adenocarcinomas and malignant carcinoid tumors of the small intestine. *Cancer Causes Control* 2013;24(9):1737-46.

6. Boffetta P, Hazelton W, Chen Y, Sinha R, Inoue M, Gao YT, et al. Body mass, tobacco smoking, alcohol drinking and risk of cancer of the small intestine-a pooled analysis of over 500,000 subjects in the Asia Cohort Consortium. *Ann Oncol* 2011;23(7):1894-98.

7. Samanic C, Chow WH, Gridley G, Jarvholm B, Fraumeni JF Jr. Relation of body mass index to cancer risk in 362,552 Swedish men. *Cancer Causes Control* 2006;17(7):901-09.

8. Bjørge T, Tretli S, Engeland A. Height and body mass index in relation to cancer of the small intestine in two million Norwegian men and women. *Br J Cancer* 2005;93(7):807-10.

9. Samanic C, Gridley G, Chow WH, Lubin J, Hoover RN, Fraumeni JF Jr. Obesity and cancer risk among white and black United States veterans. *Cancer Causes Control* 2004;15(1):35-44.
